# Tusamitamab Ravtansine in Patients with Advanced Solid Tumors: Phase I Study of Safety, Pharmacokinetics, and Antitumor Activity Using Alternative Dosing Regimens

**DOI:** 10.1158/2767-9764.CRC-23-0284

**Published:** 2023-08-28

**Authors:** Josep Tabernero, Philippe L. Bedard, Yung-Jue Bang, Maria Vieito, Min-Hee Ryu, Nathalie Fagniez, Mustapha Chadjaa, Christine Soufflet, Nina Masson, Anas Gazzah

**Affiliations:** 1 Vall d'Hebron Hospital Campus and Institute of Oncology (VHIO), UVic-UCC, IOB-Quirón, Barcelona, Spain.; 2 Division of Medical Oncology and Hematology, Department of Medicine, Princess Margaret Cancer Centre—University Health Network, University of Toronto, Toronto, Canada.; 3 Department of Internal Medicine, Seoul National University College of Medicine, Seoul, Republic of Korea.; 4 Vall d'Hebron Hospital Campus and Institute of Oncology (VHIO), Barcelona, Spain.; 5 Department of Oncology, Asan Medical Center, University of Ulsan College of Medicine, Seoul, Republic of Korea.; 6 Pharmacokinetics, Dynamics and Metabolism, Sanofi, Chilly-Mazarin, France.; 7 Clinical Development, Sanofi, Vitry-sur-Seine, France.; 8 IT&M STATS on behalf of Sanofi, Neuilly-sur-Seine, France.; 9 Department of Drug Development (DITEP), Gustave Roussy, Villejuif Cedex, Villejuif, France.

## Abstract

**Purpose:**

Tusamitamab ravtansine is an antibody–drug conjugate that targets carcinoembryonic antigen-related cell adhesion molecule 5 (CEACAM5) and delivers a cytotoxic maytansinoid payload. In a phase I dose-escalation study, the maximum tolerated dose (MTD) was 100 mg/m^2^ every 2 weeks (Q2W). Here we report results for two alternative schedules.

**Experimental Design:**

Adults ages ≥18 years (range, 34–73) with locally advanced/metastatic solid tumors (*N* = 43; colon/rectum, 29; stomach, 7; pancreas, 4; other, 3) expressing/likely to express CEACAM5 received intravenous tusamitamab ravtansine 120–170 mg/m^2^ [loading dose (LD)], then 100 mg/m^2^ Q2W (Q2W-LD, *n* = 28), or 120–190 mg/m^2^ fixed dose [every 3 weeks (Q3W), *n* = 15]. The primary endpoint was dose-limiting toxicities (DLTs) during cycles 1–2 (Q2W-LD) and cycle 1 (Q3W).

**Results:**

Reversible DLTs were observed in 2 of 9 patients (grade 2 keratopathy; grade 2 keratitis) with 170 mg/m^2^ in Q2W-LD and in 2 of 3 patients (grade 2 keratopathy; grade 3 transaminase elevation) with 190 mg/m^2^ in Q3W. Nineteen (67.9%) patients in Q2W-LD and 13 (86.7%) patients in Q3W experienced treatment-related adverse events (AE); 3 of 43 patients discontinued treatment because of AEs. The most common AEs were asthenia, gastrointestinal complaints, keratopathy, keratitis, and peripheral sensory neuropathy. In this small, heavily pretreated population, no confirmed responses were observed; however, stable disease occurred in 35.7% of patients in Q2W-LD and 40.0% of patients in Q3W.

**Conclusions:**

Tusamitamab ravtansine had a favorable safety profile with both alternative administration schedules; MTDs were 170 mg/m^2^ (LD) followed by 100 mg/m^2^ Q2W, and 170 mg/m^2^ Q3W as a fixed dose. (NCT02187848).

**Significance:**

The collective results of this phase I dose-escalation study will inform further studies of tusamitamab ravtansine in patients with solid tumors with CEACAM5 expression, including patients with non–small cell lung cancer.

## Introduction

Carcinoembryonic antigen-related cell adhesion molecule 5 (CEACAM5) is a transmembrane glycoprotein that is involved in cell adhesion and migration but has limited expression in normal adult tissues (1, 2). CEACAM5 is upregulated in several human cancers, including those of the gastrointestinal, respiratory, and genitourinary systems and the breast, and is involved in proliferation, migration, metastasis, and inhibition of apoptosis that occurs in the absence of interactions with the extracellular matrix (anoikis; refs. 3, 4). CEACAM5 is a potentially useful biomarker in patients with certain cancers (5, 6). High expression of CEACAM5 is associated with worse survival in patients with non–small cell lung cancer (NSCLC; refs. 5, 7), gastric cancer (6), and colorectal cancer (8).

Tusamitamab ravtansine (SAR408701) is an antibody–drug conjugate (ADC) designed to target tumor cells that express CEACAM5 (2). The drug consists of a humanized monoclonal antibody selective for the extracellular domain of CEACAM5, a cleavable disulfide linker, and a potent maytansinoid payload (DM4), with an average drug:antibody ratio of 3.8 (9–11). DM4 is a potent inhibitor of microtubule assembly that ultimately produces cell-cycle arrest and apoptosis. After intravenous administration, tusamitamab ravtansine binds to the extracellular domain of CEACAM5 on tumor cells and is internalized, whereupon the disulfide linker is cleaved with the release of active DM4. S-methylation of DM4 by methyltransferase also generates a highly cytotoxic moiety (S-methyl-DM4; ref. 11). In addition to their effects on CEACAM5-expressing tumor cells, both DM4 and its metabolite (S-methyl-DM4) may cross cellular membranes and produce a “bystander effect” in neighboring cells, regardless of whether those cells express CEACAM5 (11, 12).

We previously described the safety, pharmacokinetics, preliminary antitumor activity, and maximum tolerated dose (MTD) of tusamitamab ravtansine in a cohort of patients with advanced solid tumors who received escalating doses in an every 2 weeks (Q2W) schedule in the first-in-human study (NCT02187848; ref. 13). In the main dose-escalation part of the study (Q2W schedule), the MTD was determined to be 100 mg/m^2^ and the dose-limiting toxicity (DLT) was reversible keratopathy (13). Preliminary exposure response analyses of the Q2W schedule suggested that both the incidence of ocular events and the antitumor activity were correlated with exposure to tusamitamab ravtansine. A dose-expansion part of the same study assessed antitumor activity in patients with NSCLC with high or moderate CEACAM5 expression and demonstrated promising antitumor results in the high CEACAM5 expression cohort (14, 15).

Here, we describe the findings from two cohorts of patients with advanced solid tumors who received alternative dosing schedules of tusamitamab ravtansine in the same phase I clinical study. Escalating loading doses (LDs) at cycle 1, followed by fixed doses Q2W, were assessed to increase the dose/exposure in the first cycle to potentially improve efficacy while limiting the incidence of DLTs that were observed at or above the MTD in the Q2W dose-escalation part of the first-in-human study. Administration every 3 weeks (Q3W) was also assessed to align with the schedule of standard-of-care treatments at the time of the study.

## Materials and Methods

### Study Design

This was a phase I, open-label, ascending dose study in patients with advanced solid tumors (Trial registration ID: NCT02187848). The adaptive Bayesian study design, patient selection criteria, and results of the main dose-escalation cohort are published elsewhere (13). The results of two alternative dosing schedules are presented here. Dose escalation of the LD in cohort Q2W-LD and of Q3W administration in cohort Q3W was guided by an adaptive Bayesian escalation with overdose control method. The study was conducted in accordance with the Declaration of Helsinki, International Council on Harmonisation, and Good Clinical Practice guidelines. The protocol and all amendments were approved by the ethics committee or Institutional Review Board at each study site. All patients provided written informed consent before participating in the trial.

### Patients

Patient selection criteria were identical to those used for the fixed-dose Q2W schedule (13). Eligible patients were ages ≥18 years, with an Eastern Cooperative Oncology Group performance status (ECOG PS) of 0 or 1, and had locally advanced/metastatic solid malignant tumors for which no standard treatment was available. Inclusion was enriched, but not restricted, to include patients with tumors likely to express CEACAM5, or who had circulating carcinoembryonic antigen (CEA) levels >5 ng/mL as determined by a local laboratory. Tumor CEACAM5 expression in archival formalin-fixed paraffin-embedded tissue specimens was retrospectively assessed by immunohistochemistry (IHC) in a central laboratory.

Patients were excluded if they were receiving concurrent cancer treatment, had previously received CEACAM5-targeted or maytansinoid-containing regimens, or had brain metastasis, poor organ function, low bone marrow reserve, or life expectancy <12 weeks.

### Treatment

**FIGURE 1:**
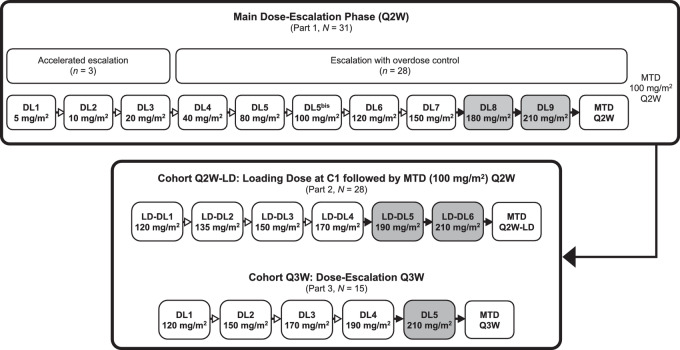
Dose-escalation schematic for the entire phase I dose-escalation study in patients with advanced solid tumors. Results of cohorts Q2W-LD and Q3W are included in the current report. The results of the main dose-escalation phase are published elsewhere (13). Dose escalation was terminated before reaching the DLs and LD-DLs shown in the shaded boxes. Numbers are the actual number of patients enrolled and treated. C1, cycle 1; DL, dose level; LD-DL, loading dose dose level; MTD, maximum tolerated dose; Q2W, every 2 weeks; Q3W, every 3 weeks.

Patients in cohort Q2W-LD received escalating LDs of tusamitamab ravtansine by intravenous infusion on day 1, cycle 1, followed by the MTD as determined in the main dose-expansion cohort (100 mg/m^2^) administered Q2W (Fig. 1). Patients in cohort Q3W received escalating doses of tusamitamab ravtansine administered Q3W. In cohort Q2W-LD, the initial LD of tusamitamab ravtansine was 120 mg/m^2^. Each LD dose level (LD-DL) in the Q2W-LD cohort and dose level (DL) in the Q3W cohort was tested sequentially in a minimum of 3 patients. Treatment was continued until disease progression, unacceptable toxicity, or willingness to stop.

To prevent hypersensitivity reactions, patients were premedicated with an oral antihistamine 1 hour prior to receiving tusamitamab ravtansine. Tusamitamab ravtansine was infused at a rate of 2.5 mg/mL for 30 minutes, then at 5 mg/mL thereafter provided there were no signs or symptoms of a hypersensitivity reaction. During the study, the protocol was amended regarding ocular prophylactic measures. Most patients in cohort Q2W-LD received primary ocular prophylaxis with an ophthalmic vasoconstrictor in both eyes before each infusion, corticosteroid preparations for 2 days starting on the day of each infusion, and use of cold masks or pads during each infusion as described previously (13); patients in cohort Q3W received secondary prophylaxis upon the recommendation of an ophthalmologist. In both cohorts, use of lubricating eye drops was encouraged.

### Endpoints

The primary endpoint was the incidence of DLTs that occurred during the first two 2-week cycles (4 weeks) in the Q2W-LD cohort and during the first 3-week cycle (3 weeks) in the Q3W cohort. The definition of a DLT used National Cancer Institute Common Terminology Criteria for Adverse Events (NCI CTCAE) version 4.03 and included hematologic toxicities (e.g., grade 4 neutropenia lasting ≥7 days, febrile neutropenia or neutropenic infection; grade 4 thrombocytopenia or grade 3 thrombocytopenia with bleeding requiring transfusion); grade ≥3 non-hematologic toxicities; grade ≥2 cardiac conduction toxicities; and any tusamitamab ravtansine-related toxicity resulting in a treatment delay of >2 weeks due to delayed recovery to baseline or grade ≤1. Ocular events were considered DLTs if they were grade ≥3 or resulted in a treatment delay of >2 weeks due to delayed recovery to baseline or grade ≤1 (13).

Secondary endpoints included the safety profile, pharmacokinetic profile, preliminary antitumor activity (per Response Evaluation Criteria in Solid Tumours [RECIST] version 1.1), and potential immunogenicity of tusamitamab ravtansine in the Q2W-LD and Q3W cohorts.

### Assessments

Safety was assessed by physical examination, laboratory test abnormalities, and treatment-emergent adverse events (TEAE). TEAEs were listed by preferred term and system organ class using the Medical Dictionary for Regulatory Activities version 23.1 and categorized by grade using NCI CTCAE version 4.03 criteria. TEAEs were considered to be serious if they were life threatening, a medically important event, or a congenital abnormality/birth defect, or resulted in death, inpatient hospitalization or prolongation of hospitalization, or persistent or significant disability/incapacity.

The pharmacokinetics of tusamitamab ravtansine in plasma were evaluated during cycle 1. A validated immunoassay (lower limit of quantitation = 0.500 μg/mL) that quantifies conjugated antibody carrying at least one DM4 payload was used to determine plasma drug concentrations (11). Pharmacokinetic parameters were calculated by noncompartmental methods.

Tumor assessments were carried out according to RECIST version 1.1 criteria at baseline, at the end of every 4 cycles (for cohort Q2W-LD) and every 2 cycles (for cohort Q3W), and at the end of treatment.

### Statistical Analyses

All analyses were descriptive and, unless otherwise specified, were conducted on the all-treated/safety population, which was defined as all patients who received at least one dose of study medication. The DLT-evaluable populations comprised all patients in cohort Q2W-LD who completed cycle 2 and who received at least 80% of the intended dose in the first two infusions and all patients in cohort Q3W who completed cycle 1 and who received at least 80% of the first infusion, unless the patient discontinued treatment because of a DLT.

### Data Availability

Qualified researchers may request access to patient-level data and related study documents, including the clinical study report, study protocol with any amendments, blank case report form, statistical analysis plan, and dataset specifications. Patient-level data will be anonymized, and study documents will be redacted to protect the privacy of our trial participants. Further details on Sanofi's data sharing criteria, eligible studies, and process for requesting access can be found at https://www.vivli.org.

## Results

### Patient Characteristics and Treatment

In cohort Q2W-LD, 38 patients were screened for eligibility and 28 patients were enrolled and treated with tusamitamab ravtansine across four LD-DLs ranging from 120 to 170 mg/m^2^ between March 13, 2017, and February 17, 2020, at study sites in Canada, France, Republic of Korea, and Spain (Table 1). In cohort Q3W, 21 patients were screened for eligibility and 15 patients were enrolled and initiated treatment with tusamitamab ravtansine across four DLs ranging from 120 to 190 mg/m^2^ between July 15, 2019, and October 20, 2020, at study sites in Canada, France, and Spain (Table 1). Reasons for screen failure are shown in Supplementary Table S1.

**TABLE 1:** Baseline demographics and disease characteristics by DL in cohorts Q2W-LD and Q3W (all-treated/safety population)

	Cohort Q2W-LD, dose of tusamitamab ravtansine (mg/m^2^)					Cohort Q3W, dose of tusamitamab ravtansine (mg/m^2^)				
Characteristic	120 C1, then 100 Q2W (*n* = 3)	135 C1, then 100 Q2W (*n* = 4)	150 C1, then 100 Q2W (*n* = 8)	170 C1, then 100 Q2W (*n* = 13)	All (*N* = 28)	120 Q3W (*n* = 3)	150 Q3W (*n* = 3)	170 Q3W (*n* = 6)	190 Q3W (*n* = 3)	All (*N* = 15)
Age, years, median (range)	59 (40–72)	65.5 (60–72)	59 (37–70)	59 (36–73)	59.5 (36–73)	59 (49–64)	64 (57–65)	60 (47–69)	52 (34-54)	59.0 (34–69)
Male sex, *n* (%)	2	4	5	8	19 (67.9)	2	3	3	0	8 (53.3)
ECOG PS, *n* (%)										
0	2	1	3	6	12 (42.9)	2	1	3	3	9 (60.0)
1	1	3	5	7	16 (57.1)	1	2	3	0	6 (40.0)
Body surface area, m^2^, median (range)	1.99 (1.5–2.1)	1.74 (1.6–2.0)	1.92 (1.8–2.4)	1.76 (1.6–2.1)	1.84 (1.5–2.4)	1.82 (1.6–1.9)	1.95 (1.9–2.2)	1.63 (1.5–2.1)	1.77 (1.5–1.9)	1.80 (1.5–2.2)
Measurable disease, *n* (%)	3	3	8	13	27 (96.4)	3	3	6	3	15 (100)
Primary tumor location, *n* (%)										
Lung	0	0	0	1	1 (3.6)	0	0	0	0	0
Colon/rectum	3	1	6	10	20 (71.4)	0	3	3	3	9 (60.0)
Stomach	0	2	2	2	6 (21.4)	0	0	1	0	1 (6.7)
Gastroesophageal junction	0	1	0	0	1 (3.6)	1	0	0	0	1 (6.7)
Pancreas	0	0	0	0	0	2	0	2	0	4 (26.7)
Organs involved^a^ in >30% patients in either cohort, *n* (%)										
Lung	3	2	4	8	17 (60.7)	1	3	3	2	9 (60.0)
Liver	1	1	5	9	16 (57.1)	2	1	4	2	9 (60.0)
Lymph node	1	1	4	9	15 (53.6)	2	1	4	2	9 (60.0)
Number of prior regimens, median (range)	5 (3–5)	2.5 (2–4)	4 (2–9)	5 (3–6)	4 (2–9)	4 (4–5)	5 (3–5)	2 (2–5)	3 (2–5)	3 (2–5)
Prior anti-tubulin exposure, *n* (%)	0	3	1	3	7 (25.0)	2	0	2	0	4 (26.7)
CEACAM5 expression in tumor samples,^b^*n* (%)										
Patients tested, *n*	3	4	7	13	27	2	2	2	2	8
<50%	1	1	2	5	9 (33.3)	0	0	1	1	2 (25.0)
50%–80%	1	0	1	3	5 (18.5)	1	0	1	0	2 (25.0)
≥80%	1	3	4	5	13 (48.1)	1	2	0	1	4 (50.0)
Circulating CEA level, *n* (%)										
Patients tested, *n*	3	4	8	13	28	3	3	6	2	14
<5 μg/L	1	2	2	2	7 (25.0)	0	0	0	0	0
≥5 μg/L	2	2	6	11	21 (75.0)	3	3	6	2	14 (100)

Abbreviations: C1, cycle 1; CEA, carcinoembryonic antigen; CEACAM5, carcinoembryonic antigen-related cell adhesion molecule 5; ECOG PS, Eastern Cooperative Oncology Group performance status; Q2W, every 2 weeks; Q2W-LD, cohort receiving a loading dose at day 1, cycle 1, followed by a fixed dose every 2 weeks; Q3W, cohort receiving tusamitamab ravtansine every 3 weeks; Q3W, every 3 weeks; RECIST, Response Evaluation Criteria in Solid Tumours.

^a^Organs involved include target or non-target lesions as defined by RECIST version 1.1 reported by the investigators at baseline.

^b^At intensity 2+/3+ (on archival sample).

As of the date of publication, all patients in cohort Q2W-LD and in cohort Q3W had discontinued treatment. In cohort Q2W-LD, 26 patients discontinued treatment because of disease progression and 2 because of adverse events (AEs), and in cohort Q3W, 14 patients discontinued treatment because of disease progression and 1 because of AEs.

The median ages of patients in cohorts Q2W-LD and Q3W were 59.5 and 59.0 years, respectively, and a majority were male (67.9% and 53.3%, respectively; Table 1). The representativeness of the patient population is described in Supplementary Table S2. All patients in cohorts Q2W-LD and Q3W had metastatic disease, and the median number of prior anticancer treatment regimens was 4 (range, 2–9) and 3 (2–5), respectively. CEACAM5 expression at intensity 2+/3+ in <50%, 50%–80%, and ≥80% of tumor cells was observed in 33.3%, 18.5%, and 48.1% of patients in cohort Q2W-LD and 25.0%, 25.0%, and 50.0% of patients in cohort Q3W, respectively. The most common primary tumor location at initial diagnosis was the colon/rectum (71.4% and 60.0% in cohorts Q2W-LD and Q3W, respectively), and the most common involved organs were lung (60.7%) in cohort Q2W-LD and liver, lung, and lymph node (60.0% each) in cohort Q3W.

In cohort Q2W-LD, a total of 128 cycles were administered across all DLs (Supplementary Table S3). Overall, median duration of treatment was 8 weeks (range, 2–32). Among the 13 patients in the highest LD level (170 mg/m^2^), the median duration of treatment was 8 weeks (range, 2–32), the median number of cycles administered was 4 per patient (range, 1–12), and the median cumulative dose was 469 mg/m^2^ (range, 172–1,190 mg/m^2^). In the entire Q2W-LD cohort, 12 (42.9%) patients underwent at least one dose modification due to a TEAE, including 8 of 13 patients in the highest LD-DL (170 mg/m^2^).

At the time of study cutoff, in cohort Q3W, a total of 39 cycles were administered across all DLs (Supplementary Table S3). The median duration of treatment was 6.6 weeks (range, 3–18). Among the 3 patients at the highest dose (190 mg/m^2^) in cohort Q3W, the median duration of treatment was 6.0 weeks (range, 3.0–8.3), the median number of cycles administered was 2 per patient (range, 1–2), and the median cumulative dose was 354 mg/m^2^ (range, 188–382 mg/m^2^). Seven (46.7%) patients underwent dose modifications due to a TEAE, including 5 of 6 patients who received 170 mg/m^2^ and 1 of 3 patients who received 190 mg/m^2^.

### DLTs

In cohort Q2W-LD, the DLT-evaluable population comprised 21 of 28 patients, including 3, 3, 6, and 9 patients at LD-DLs 120, 135, 150, and 170 mg/m^2^, respectively. Reasons for exclusion from the DLT population included early discontinuation for disease progression (1 patient at 135 mg/m^2^ and 2 patients at 170 mg/m^2^), receipt of a second LD at cycle 2, day 1 due to a dosing error (2 patients at 170 mg/m^2^), receipt of ≤80% of the intended dose during the second infusion due to drug hypersensitivity (1 patient at 150 mg/m^2^), and patient deterioration prior to the end of the DLT period (1 patient at 150 mg/m^2^).

Two of 9 DLT-evaluable patients experienced a DLT at the 170 mg/m^2^ DL. One patient experienced grade 2 keratopathy during cycle 2, had a treatment delay, and then resumed treatment at a reduced dose; the keratopathy resolved after 33 days. The second patient experienced grade 2 keratitis during cycle 2 and withdrew from therapy; the keratitis resolved after 31 days. No DLTs were observed in patients receiving tusamitamab ravtansine at lower LD-DLs (120, 135, and 150 mg/m^2^).

In cohort Q3W, all 15 patients were evaluable for DLTs. Two of the 3 DLT-evaluable patients at the 190 mg/m^2^ DL experienced a DLT. One patient experienced grade 2 keratopathy during cycle 1 and recovered after a treatment delay of 59 days. Treatment was resumed at a reduced dose after the cycle delay. The second patient experienced a grade 3 increase in transaminase levels during cycle 1, which prompted withdrawal of therapy; the transaminase elevation resolved after 19 days. No DLTs were observed in patients receiving tusamitamab ravtansine at lower DLs (120, 150, or 170 mg/m^2^).

On the basis of these observations, the MTD was defined as a LD of 170 mg/m^2^ followed by 100 mg/m^2^ Q2W in cohort Q2W-LD, and 170 mg/m^2^ Q3W as a fixed dose in cohort Q3W.

### Safety

All patients in cohorts Q2W-LD and Q3W experienced ≥1 TEAE (Table 2). Treatment-related TEAEs were reported in 19 of 28 (67.9%) patients in cohort Q2W-LD and included keratopathy in 5 (17.9%) patients; keratitis, dry eye, peripheral sensory neuropathy, and asthenia in 4 (14.3%) patients each; nausea in 3 (10.7%) patients; decreased appetite, diarrhea, abdominal pain, pyrexia, and accidental overdose of study medication in 2 (7.1%) patients each; and drug hypersensitivity, neurotoxicity, paresthesia, ocular exfoliation syndrome, eye pruritus, foreign-body sensation in the eyes, blurred vision, flushing, dysphonia, vomiting, general illness, and decreased platelet count in 1 (3.6%) patient each. Four (14.3%) patients in cohort Q2W-LD who received the 170 mg/m^2^ LD had grade 3–4 treatment-related TEAEs [keratopathy (*n* = 2), keratitis (*n* = 1), and decreased platelet count (*n* = 1)]. No patients in cohort Q2W-LD had a serious treatment-related TEAE. In cohort Q3W, 13 of 15 (86.7%) patients had treatment-related TEAEs, including asthenia in 4 (26.7%) patients; peripheral sensory neuropathy, keratopathy, and keratitis in 3 (20.0%) patients each; decreased appetite, paresthesia, abdominal pain, nausea, and fatigue in 2 (13.3%) patients each; and diarrhea, myalgia, increased transaminases, and infusion-related reaction in 1 (6.7%) patient each. Two of 15 (13.3%) patients in cohort Q3W had treatment-related TEAEs of grade 3–4 (keratopathy in 1 patient at the 170 mg/m^2^ DL and increased transaminases in 1 patient at the 190 mg/m^2^ DL). One of 6 (16.7%) patients at the 170 mg/m^2^ DL in cohort Q3W had a serious treatment-related TEAE (grade 2 drug infusion-related reaction during cycle 1).

**TABLE 2:** Treatment-emergent adverse events in cohorts Q2W-LD and Q3W (all-treated/safety population)

	Cohort Q2W-LD, dose of tusamitamab ravtansine (mg/m^2^)					Cohort Q3W, dose of tusamitamab ravtansine (mg/m^2^)				
Patients with TEAE, *n* (%)	120 C1, then 100 Q2W (*n* = 3)	135 C1, then 100 Q2W (*n* = 4)	150 C1, then 100 Q2W (*n* = 8)	170 C1, then 100 Q2W (*n* = 13)	All (*N* = 28)	120 Q3W (*n* = 3)	150 Q3W (*n* = 3)	170 Q3W (*n* = 6)	190 Q3W (*n* = 3)	All (*N* = 15)
Any TEAE	3	4	8	13	28 (100)	3	3	6	3	15 (100)
Any treatment-related TEAE	0	3	6	10	19 (67.9)	2	2	6	3	13 (86.7)
Treatment-related grade ≥3 TEAE	0	0	0	4	4 (14.3)	0	0	1	1	2 (13.3)
Serious treatment-related TEAE	0	0	0	0	0	0	0	1	0	1 (6.7)
TEAE leading to dose modification	1	0	3	8	12 (42.9)	1	0	5	1	7 (46.7)
TEAE leading to discontinuation	0	0	1	1	2 (7.1)	0	0	0	1	1 (6.7)
TEAE occurring in ≥10% of patients in either cohort,^a^										
Asthenia	0	1	1	4	6 (21.4)	1	1	1	1	4 (26.7)
Nausea	1	0	1	4	6 (21.4)	0	0	2	1	3 (20.0)
Abdominal pain	0	1	2	2	5 (17.9)	0	0	3	0	3 (20.0)
Keratopathy	0	0	1	4	5 (17.9)	0	0	2	1	3 (20.0)
Diarrhea	0	2	2	0	4 (14.3)	0	0	1	0	1 (6.7)
Dry eye	0	0	2	2	4 (14.3)	—	—	—	—	—
Dyspnea	2	1	0	1	4 (14.3)	—	—	—	—	—
Keratitis	0	0	0	4	4 (14.3)	1	0	2	0	3 (20.0)
Peripheral sensory neuropathy	0	1	1	2	4 (14.3)	0	2	1	0	3 (20.0)
Cough	1	1	0	1	3 (10.7)	—	—	—	—	—
Decreased appetite	1	0	0	2	3 (10.7)	1	0	1	1	3 (20.0)
Fatigue	1	1	1	0	3 (10.7)	0	0	0	2	2 (13.3)
Constipation	0	0	1	0	1 (3.6)	1	1	1	1	4 (26.7)
Paresthesia	0	0	0	1	1 (3.6)	0	0	1	1	2 (13.3)
Pyrexia	0	0	1	1	2 (7.1)	0	1	1	0	2 (13.3)

Abbreviations: C1, cycle 1; Q2W, every 2 weeks; Q2W-LD, cohort receiving a loading dose at day 1, cycle 1, followed by a fixed dose every 2 weeks; Q3W, cohort receiving tusamitamab ravtansine every 3 weeks; Q3W, every 3 weeks; TEAE, treatment-emergent adverse event.

^a^Any grade, regardless of relationship to treatment.

Two patients in cohort Q2W-LD and 1 patient in cohort Q3W discontinued treatment because of TEAEs (Table 2). TEAEs leading to treatment discontinuation included keratitis in a patient during cycle 2 at the 170 mg/m^2^ LD-DL and sudden death unrelated to treatment during cycle 7 at the 150 mg/m^2^ LD-DL in cohort Q2W-LD, and increased transaminases during cycle 1 at the 190 mg/m^2^ DL in cohort Q3W.

Corneal events were a prominent TEAE in patients in both cohorts. In cohort Q2W-LD, 9 of 28 (32.1%) patients experienced corneal events, all occurring at the two highest LD-DLs (1 at 150 mg/m^2^ and 8 at 170 mg/m^2^), including keratopathy in 5 patients (2 with grade 3) and keratitis in 4 patients (1 with grade 3). The first occurrence was at cycle 2 for 7 patients and at cycle 4 for 2 patients. Corneal events were managed by dose modifications in 8 patients, including cycle delays (*n* = 8) or cycle delays in combination with dose reductions (*n* = 2), and resolved after a median of 32 days (range, 9–59 days) in 8 of 9 patients. Five patients experienced one corneal event each, 2 patients experienced two events each, and 2 patients experienced three events each.

In cohort Q3W, 6 of 15 (40.0%) patients experienced corneal events (1 at DL 120 mg/m^2^, 4 at DL 170 mg/m^2^, and 1 at DL 190 mg/m^2^), including 3 patients with keratopathy (1 grade 3) and 3 patients with keratitis (all grade <3). For these 6 patients, the first occurrence was observed at cycle 1 (*n* = 3), cycle 2 (*n* = 1), cycle 3 (*n* = 1), and during follow-up (*n* = 1), and the events were managed by cycle delays (*n* = 4) or by cycle delay and dose reduction (*n* = 1); treatment was not modified for the other patient. Corneal events resolved in 3 patients within a median time of 38 days (range, 8–59 days). One patient was recovering, and 2 had not recovered at the time of data cutoff. Three patients experienced one event each, and 3 patients experienced two events each.

In cohorts Q2W-LD and Q3W, 4 and 3 patients, respectively, experienced grade 1 peripheral sensory neuropathy that did not require dose modifications. None of the 4 patients in cohort Q2W-LD but all 3 patients in cohort Q3W had a history of peripheral sensory neuropathy.

In cohort Q2W-LD, 8 of 28 patients died during the study, and in cohort Q3W, 3 of 15 patients died. No deaths were considered to be related to treatment. Reasons for death in cohort Q2W-LD included disease progression (*n* = 7) and other reason (*n* = 1, sudden death with symptoms that suggested a massive thrombotic event). All deaths in cohort Q3W were attributed to disease progression.

### Antitumor Activity

No patients in cohorts Q2W-LD or Q3W experienced a confirmed partial or complete response. In cohort Q2W-LD overall, 10 patients had stable disease (time to progression range: 1.5–7.2 months; 5 patients ≥4 months), 15 patients had progressive disease, and 3 patients were not evaluable (no post-baseline evaluation). Six of 13 patients who received a LD of 170 mg/m² had stable disease. In cohort Q3W overall, 6 patients had stable disease (time to progression range: 1.2–2.8 months), 7 patients had progressive disease, and 2 patients were not evaluable. Two of 6 patients who received 170 mg/m² Q3W had stable disease.

### Pharmacokinetics

**FIGURE 2:**
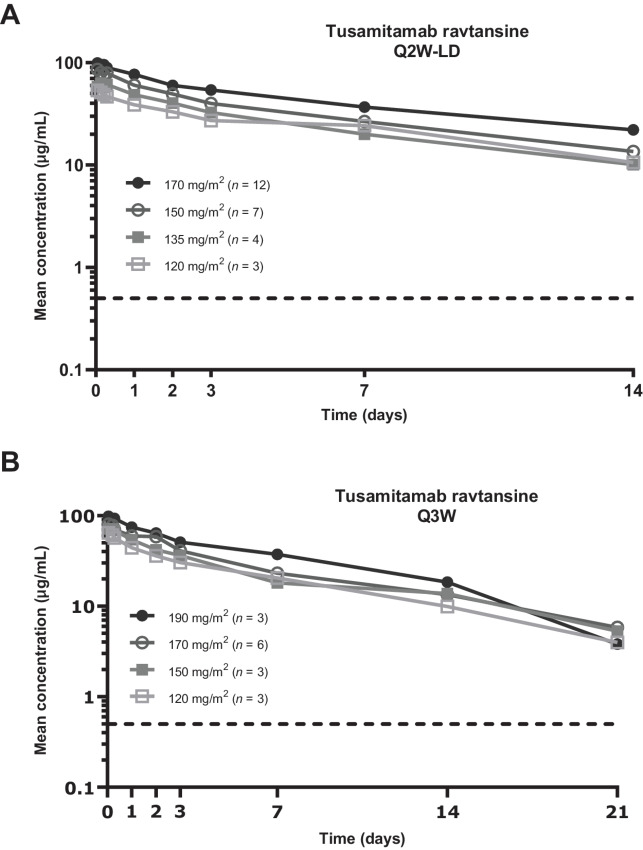
Pharmacokinetic profile of tusamitamab ravtansine during the first cycle of treatment in patients with advanced solid tumors. Plasma concentration over time is shown for patients receiving an LD (120–170 mg/m^2^) followed by 100 mg/m^2^ every 2 weeks (cohort Q2W-LD, **A**) and for patients receiving fixed doses (120–190 mg/m^2^) every 3 weeks (cohort Q3W, **B**). The dotted line indicates the lower limit of quantitation (0.5 μg/mL). Q2W-LD, cohort receiving tusamitamab ravtansine as a loading dose at day 1, cycle 1, followed by a fixed dose every 2 weeks; Q3W, cohort receiving tusamitamab ravtansine every 3 weeks.

The pharmacokinetic-evaluable population for tusamitamab ravtansine included 26 patients from cohort Q2W-LD and 15 patients from cohort Q3W. After the first administration, tusamitamab ravtansine plasma concentrations remained quantifiable up to day 14 in the Q2W-LD cohort (Fig. 2A) and up to day 21 in the Q3W cohort (Fig. 2B).

Exposure to tusamitamab ravtansine (maximum plasma concentration, *C*_max_, and area under the plasma concentration vs. time curve, AUC) increased in a slightly more than dose-proportional manner after administration of LDs (120–170 mg/m²) in cohort Q2W-LD (Supplementary Figs. S1A and S2A; Supplementary Table S4) and was approximately dose proportional after administration of the first dose (120–190 mg/m²) in cohort Q3W (Supplementary Figs. S1B and S2B; Supplementary Table S5). Variability in exposure, as indicated by the coefficient of variation (CV%), was low to moderate for *C*_max_ (range, 18%–36% in cohort Q2W-LD and 5%–30% in cohort Q3W) and AUC (range, 12%–52% in cohort Q2W-LD and 20%–33% in cohort Q3W). Mean (CV%) *C*_max_ and AUC were 101 μg/mL (18%) and 773 μg•day/mL (32%), respectively, after administration of the 170 mg/m² LD in cohort Q2W-LD, and 84.7 μg/mL (30%) and 590 μg•day/mL (28%), respectively, after administration of 170 mg/m^2^ in the first cycle in cohort Q3W.

Clearance ranged from approximately 0.4 to 0.7 L/day, and the terminal elimination half-life ranged from approximately 6 to 8 days across all DLs in cohorts Q2W-LD and Q3W (Supplementary Tables S4 and S5).

### Immunogenicity

At data cutoff, 4 of 25 (16%) evaluable patients in cohort Q2W-LD and no patients among the 13 evaluable patients in cohort Q3W had treatment-induced antitherapeutic antibodies.

## Discussion

The results from cohorts Q2W-LD and Q3W of this phase I dose-escalation trial in patients with advanced solid tumors confirm and extend the understanding of the DLTs, safety, and pharmacokinetics of tusamitamab ravtansine established in the earlier first-in-human main dose-escalation study results (13). Tusamitamab ravtansine had an acceptable safety profile when administered in a LD regimen (120–170 mg/m^2^ followed by 100 mg/m^2^ Q2W) and in a fixed-dose regimen (120–170 mg/m^2^ Q3W). DLTs included corneal events (grade 2 keratopathy and keratitis) in both the Q2W-LD and Q3W cohorts, and increased transaminases in 1 patient in the Q3W cohort. Dose-related keratopathy was also identified as the DLT in the previously reported main dose-escalation cohort for tusamitamab ravtansine Q2W without a LD, in which the MTD was identified as 100 mg/m^2^ (13). All DLTs in the current study were nonfatal and reversible after dose modification (cycle delays or dose reductions).

The most common TEAEs were asthenia, nausea, abdominal pain, and keratopathy in both cohorts. Grade 1 peripheral neuropathy was also observed in both cohorts [incidence 21.4% in the Q2W-LD cohort (*n* = 4 peripheral sensory neuropathy, *n* = 1 paresthesia, *n* = 1 neurotoxicity) and 33.3% in the Q3W cohort (*n* = 3 peripheral sensory neuropathy, *n* = 2 paresthesia)]. Dose discontinuations for TEAEs were implemented infrequently (<10% of patients) in both cohorts.

Prophylactic measures were used during the main Q2W (13) and Q2W-LD dose-escalation cohorts in an attempt to prevent or mitigate corneal AEs; however, primary prophylaxis with a vasoconstrictor and corticosteroid did not appear to impact the occurrence of corneal events, consistent with a separate analysis of patients from dose-expansion cohorts of the study. Hence, primary corneal prophylaxis is no longer recommended to patients enrolled in clinical trials of tusamitamab ravtansine. Rather, secondary prophylaxis is being considered on the recommendation of an ophthalmologist.

A variety of ocular AEs have been reported with a range of ADCs that have diverse biological targets and cytotoxic payloads (16–18). Corneal AEs have been associated with ADCs that have microtubule inhibitors as payloads (auristatin or maytansinoid derivatives), are DLTs for several compounds, and are a frequent cause of dose modifications for some agents (19–27). As a result, patients with a history of corneal disease have been excluded from phase III clinical trials of some ADCs [e.g., belantamab mafodotin (28), enfortumab vedotin (29), and trastuzumab deruxtecan (30)]. Administration of prophylactic corticosteroids has been reported to be ineffective in preventing corneal AEs in these studies (28, 31); thus, dose modifications are the preferred management strategy to minimize ocular toxicity for several ADCs (16, 26, 29).

In a phase II trial in patients with multiple myeloma, severe (grade 3/4) corneal AEs were reported in 46% of patients treated with an ADC (belantamab mafodotin) that targets B-cell maturation antigen, a marker that is expressed exclusively on malignant plasma cells (28, 32). The mechanism by which belantamab mafodotin causes corneal toxicity is unknown, although it has been speculated that it is taken up by corneal progenitor cells via macropinocytosis, primarily through an off-target mechanism (32). It remains to be determined whether a similar process could be involved in ocular toxicity associated with tusamitamab ravtansine.

No patients in either cohort had objective responses, although stable disease was observed in 10 of 25 (40.0%) evaluable patients in cohort Q2W-LD and 6 of 13 (46.2%) evaluable patients in cohort Q3W. It is unclear why objective responses were not observed with the dosing schedules in the current study, although the heavily pretreated, and potentially refractory, patient population in this study may have been a contributing factor. Ongoing clinical trials are evaluating responses to tusamitamab ravtansine treatment in patients with NSCLC, gastric cancer, or pancreatic cancer and may provide additional insight. Partial responses were observed in 3 patients enrolled in the main dose-escalation cohort who were treated with 100 or 120 mg/m^2^ Q2W (13). Two of the 3 patients with partial responses in the main Q2W dose-escalation cohort reported previously (13) had colorectal cancer, which was the most common primary tumor in patients enrolled in the current study. In addition, 2 of the 3 patients with a partial response in the main Q2W dose-escalation cohort (13) had strong expression of CEACAM5 (intensity ≥2+ in 100% of tumor cells), whereas 17 of 43 (40%) patients in the current study had CEACAM5 expression of intensity ≥2+ in ≥80% of tumor cells. In a subsequent dose-expansion study in patients with heavily pretreated NSCLC, 20% of patients with high CEACAM5 expression (intensity ≥2+ in ≥50% of tumor cells) had a partial response, and almost half of those patients maintained the response for 1 year (14, 15). Predictors of response will be examined more closely in future efficacy studies.

Consistent with observations in the main Q2W dose-escalation cohort, exposure (C_max_ and AUC) to tusamitamab ravtansine increased in a dose-proportional manner over the dose ranges examined with no dose effect on clearance (13).

The collective results of the dose-escalation cohorts presented in the present and previous analyses have practical implications for the clinical development of tusamitamab ravtansine. The drug exposure profile and MTD have now been determined for three different regimens, and the DLT of tusamitamab ravtansine was consistent in each cohort. This provides flexibility when designing dosing schedules for use in future trials and in combination with other agents. To this end, the results of this study have informed the design of further studies of tusamitamab ravtansine in patients with solid tumors. Ongoing trials are investigating the safety and efficacy of tusamitamab ravtansine Q2W as monotherapy (CARMEN-LC03; NCT04154956), Q2W in combination with ramucirumab (CARMEN-LC04; NCT04394624), and Q3W in combination with pembrolizumab (CARMEN-LC05; NCT04524689) in patients with NSCLC; and Q2W-LD in patients with advanced gastric or gastroesophageal junction adenocarcinoma (CARMEN-GC01; NCT05071053; refs. 33–36).

In conclusion, the MTD of tusamitamab ravtansine was 170 mg/m^2^ when administered as a LD followed by a 100 mg/m^2^ dose Q2W in cohort Q2W-LD and when administered as a fixed dose Q3W in cohort Q3W. The DLTs included keratopathy and keratitis in the Q2W-LD cohort and keratopathy and increased transaminases in the Q3W cohort. Consistent with the main dose-escalation cohort, the most frequent TEAEs were corneal events, which occurred at rates similar to those in patients who received tusamitamab ravtansine 100 mg/m^2^ Q2W in the main dose-escalation cohort.

## Supplementary Material

Supplemental Figure S1 Tusamitamab ravtansine maximum plasma concentrations by dose level

Supplemental Figure S2 Tusamitamab ravtansine area under the curve by dose level

Supplemental Table S1 Reasons for screen failure

Supplemental Table S2 Representativeness of the patient population

Supplemental Table S3 Intensity of treatment

Supplemental Table S4 Plasma pharmacokinetics of tusamitamab ravtansine (cohort Q2W-LD)

Supplemental Table S5 Plasma pharmacokinetics of tusamitamab ravtansine (cohort Q3W)
