# Supplementary material for: Tusamitamab Ravtansine in Patients with Advanced Solid Tumors: Phase I Study of Safety, Pharmacokinetics, and Antitumor Activity Using Alternative Dosing Regimens
Source: Cancer Res Commun. 2023 Aug 28;3(8):1662–71. doi: 10.1158/2767-9764.CRC-23-0284 (PMC10461573; doi:10.1158/2767-9764.CRC-23-0284)

Supplementary Figure S1. Individual and mean (± standard deviation) tusamitamab ravtansine maximum plasma concentrations by dose level after first administration in Cohort Q2W-LD (A) and Cohort Q3W (B).

Cmax, maximum plasma concentration; Q2W-LD, cohort receiving a loading dose at Day 1, Cycle 1, followed by a fixed dose every 2 weeks; Q3W, cohort receiving tusamitamab ravtansine every 3 weeks; SD, standard deviation.

A

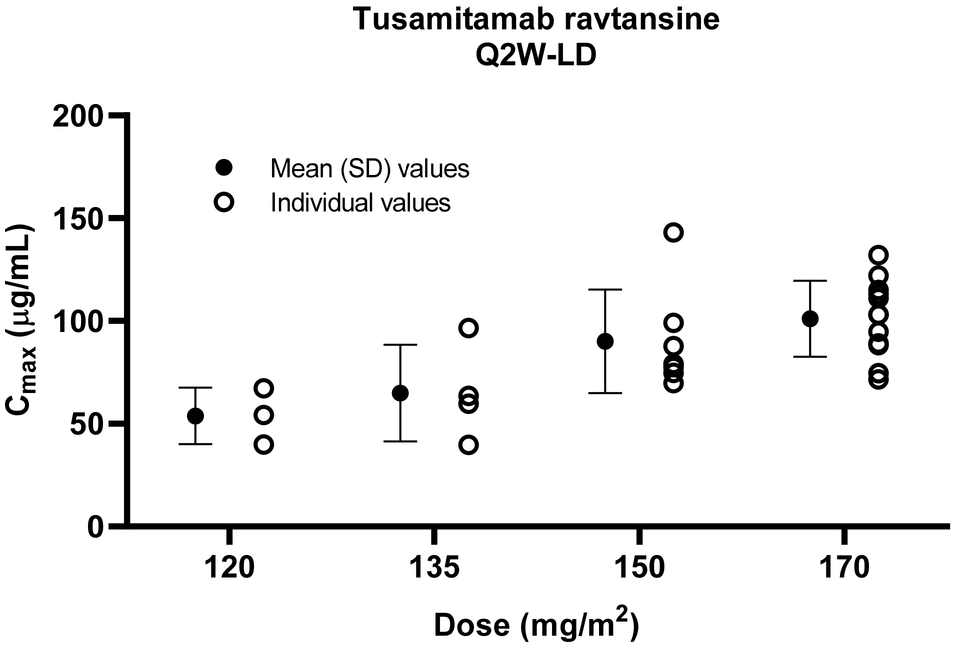

B

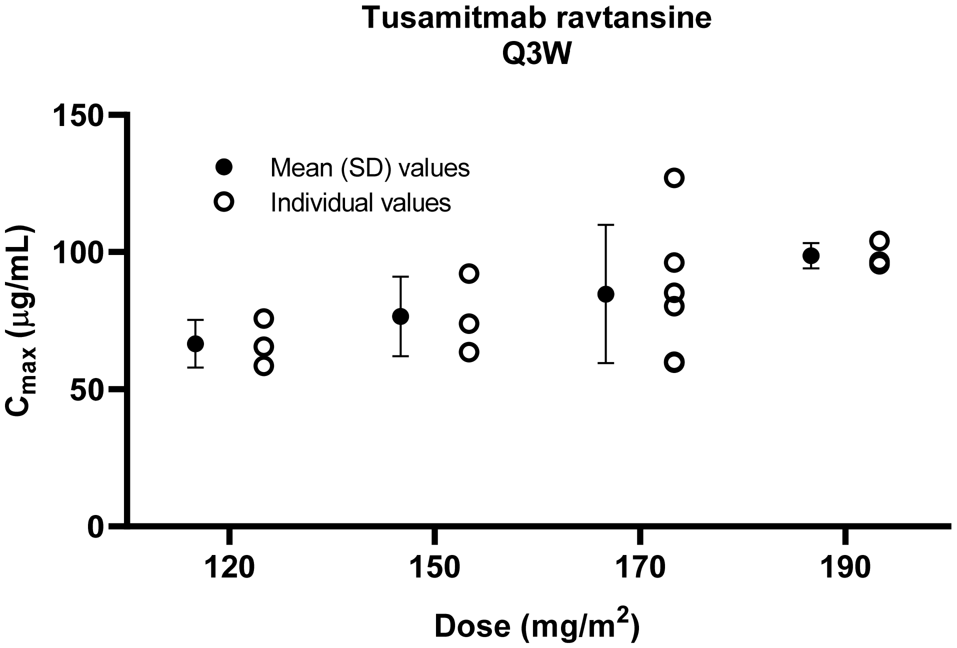

Supplement: Supplemental Figure S1 — Tusamitamab ravtansine maximum plasma concentrations by dose level [file crc-23-0284-s01.pdf]
