# Supplementary material for: Tusamitamab Ravtansine in Patients with Advanced Solid Tumors: Phase I Study of Safety, Pharmacokinetics, and Antitumor Activity Using Alternative Dosing Regimens
Source: Cancer Res Commun. 2023 Aug 28;3(8):1662–71. doi: 10.1158/2767-9764.CRC-23-0284 (PMC10461573; doi:10.1158/2767-9764.CRC-23-0284)

Supplementary Figure S2. Individual and mean (± standard deviation) area under the curve of tusamitamab ravtansine by dose level after first administration in Cohort Q2W-LD (A) and Cohort Q3W (B).
AUC, area under the plasma concentration versus time curve from time zero extrapolated to infinity; Q2W-LD, cohort receiving a loading dose at Day 1, Cycle 1, followed by a fixed dose every 2 weeks; Q3W, cohort receiving tusamitamab ravtansine every 3 weeks; SD, standard deviation.

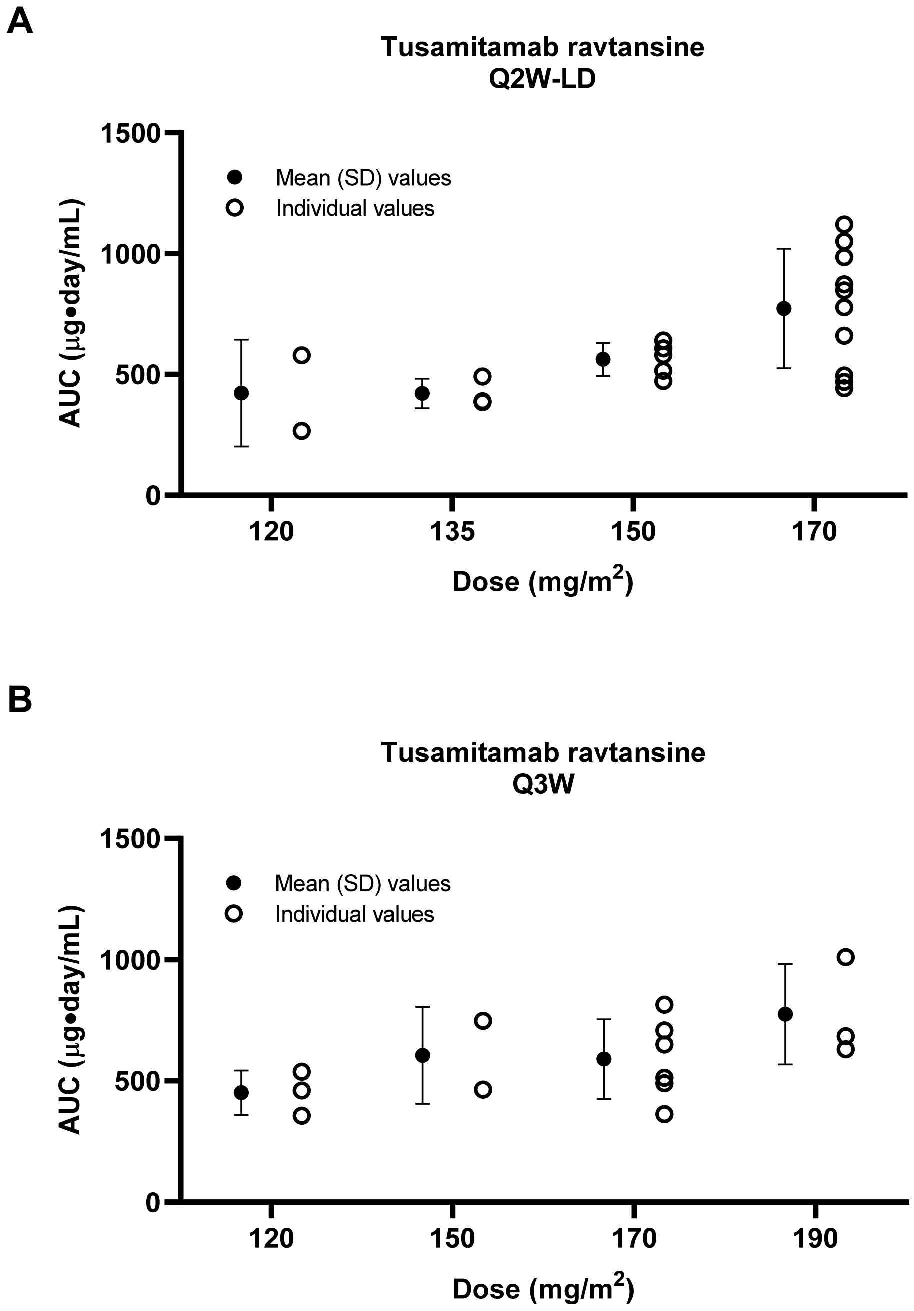

Supplement: Supplemental Figure S2 — Tusamitamab ravtansine area under the curve by dose level [file crc-23-0284-s02.pdf]
