# Supplementary material for: Tusamitamab Ravtansine in Patients with Advanced Solid Tumors: Phase I Study of Safety, Pharmacokinetics, and Antitumor Activity Using Alternative Dosing Regimens
Source: Cancer Res Commun. 2023 Aug 28;3(8):1662–71. doi: 10.1158/2767-9764.CRC-23-0284 (PMC10461573; doi:10.1158/2767-9764.CRC-23-0284)
Supplement: Supplemental Table S1 — Reasons for screen failure [file crc-23-0284-s03.pdf]

Supplementary Table S1. Reasons for screen failure in cohorts Q2W-LD and Q3W

	Cohort Q2W-LD	Cohort Q3W
Patients screened, n	38	21
Screen failures, n	10	6
Reasons for screen failure, n[a]		
No available FFPE archival tissue to evaluate CEACAM5 expression	0	3
ECOG PS > 1	1	0
Unwillingness or inability to comply with study procedures	1	0
Significant concomitant illness	3	2
Significant laboratory abnormalities: SCr > 1.5× ULN or 1.0–1.5× ULN with CrCl < 60 mL/min; total bilirubin > 1.5× ULN; and/or AST or ALT > 2.5× ULN (without liver metastasis) or > 5× ULN (with liver metastasis)	1	2
Previous history of and/or unresolved corneal disorders or use of contact lenses	1	2
Clinically significant cardiac conduction disturbances/arrhythmias	1	0
Contraindications to the use of topical ophthalmic vasoconstrictors and/or corticosteroids	2	0

[a]More than one criterion could apply for an individual patient.

ALT, alanine aminotransferase; AST, aspartate aminotransferase; CEACAM5, carcinoembryonic antigen-related cell adhesion molecule 5; CrCl, creatinine clearance; ECOG PS, Eastern Cooperative Oncology Group performance status; FFPE, formalin-fixed paraffin-embedded; Q2W-LD, cohort receiving a loading dose at Day 1, Cycle 1, followed by a fixed dose every 2 weeks; Q3W, cohort receiving tusamitamab ravtansine every 3 weeks; SCr, serum creatinine; ULN, upper limit of normal.
