# Supplementary material for: Tusamitamab Ravtansine in Patients with Advanced Solid Tumors: Phase I Study of Safety, Pharmacokinetics, and Antitumor Activity Using Alternative Dosing Regimens
Source: Cancer Res Commun. 2023 Aug 28;3(8):1662–71. doi: 10.1158/2767-9764.CRC-23-0284 (PMC10461573; doi:10.1158/2767-9764.CRC-23-0284)
Supplement: Supplemental Table S2 — Representativeness of the patient population [file crc-23-0284-s04.pdf]

Supplementary Table S2. Study population representativeness

Characteristic	Comment
Sex	See Table 1. Although there were more men (67.9% of the total) than women in Cohort Q2W-LD, Cohort Q3W was roughly balanced between men (53.3%) and women.
Age	See Table 1. Cohorts Q2W-LD and Q3W had patients with a median age of 59.5 and 59.0 years, respectively. The age range across the two cohorts was broad, from 34−73 years of age.
Race/ethnicity	Most (24 of 28) patients in Cohort Q2W-LD were White, and most (*n* = 21) were non-Hispanic; 4 were Asian. All 15 patients in Cohort Q3W were White; a similar number of patients in Cohort Q3W were non-Hispanic (*n* = 8) versus Hispanic (*n* = 7).
Geography	The study was conducted at sites in 4 countries total. Patients in Cohort Q2W-LD were enrolled at study sites in Canada, France, Republic of Korea, and Spain. Patients in Cohort Q3W were enrolled at study sites in Canada, France, and Spain.
Other considerations	The fact that this is a Phase 1 study conducted at a small number of sites in 4 countries inherently limits the population representativeness.

Q2W-LD, cohort receiving a loading dose of tusamitamab ravtansine at Day 1, Cycle 1, followed by a fixed dose every 2 weeks; Q3W, cohort receiving tusamitamab ravtansine every 3 weeks
