# Supplementary material for: Tusamitamab Ravtansine in Patients with Advanced Solid Tumors: Phase I Study of Safety, Pharmacokinetics, and Antitumor Activity Using Alternative Dosing Regimens
Source: Cancer Res Commun. 2023 Aug 28;3(8):1662–71. doi: 10.1158/2767-9764.CRC-23-0284 (PMC10461573; doi:10.1158/2767-9764.CRC-23-0284)
Supplement: Supplemental Table S3 — Intensity of treatment [file crc-23-0284-s05.pdf]

Supplementary Table S3. Intensity of treatment in cohorts Q2W-LD and Q3W (all-treated/safety population)

Characteristic	Cohort Q2W-LD, dose of tusamitamab ravtansine (mg/m^2)					Cohort Q3W, dose of tusamitamab ravtansine (mg/m^2)				
	120 C1, then 100 Q2W (*n* = 3)	135 C1, then 100 Q2W (*n* = 4)	150 C1, then 100 Q2W (*n* = 8)	170 C1, then 100 Q2W (*n* = 13)	All (*N* = 28)	120 Q3W (*n* = 3)	150 Q3W (*n* = 3)	170 Q3W (*n* = 6)	190 Q3W (*n* = 3)	All (*N* = 15)
Median duration of study treatment, weeks	12.0 (6.3, 14.0)	6.1 (2.0, 27.1)	8.0 (4.0, 16.3)	8.0 (2.0, 32.1)	8.0 (2.0, 32.1)	7.0 (6.0, 12.0)	12.0 (6.0, 18.0)	6.3 (3.0, 12.9)	6.0 (3.0, 8.3)	6.6 (3.0, 18.0)
Cycles administered to all patients, *n*	15	19	31	63	128	8	12	14	5	39
Median cycles administered per patient, *n*	6.0 (3.0, 6.0)	3.0 (1.0, 12.0)	4.0 (2.0, 7.0)	4.0 (1.0, 12.0)	4.0 (1.0, 12.0)	2.0 (2.0, 4.0)	4.0 (2.0, 6.0)	2.0 (1.0, 4.0)	2.0 (1.0, 2.0)	2.0 (1.0, 6.0)
Median cumulative dose, mg/m^2	613 (316, 622)	323 (135, 1254)	448.5 (248, 742)	469 (172, 1190)	—	241 (238, 482)	564 (297, 742)	334.5 (178, 678)	354 (188, 382)	—

Values are median (minimum, maximum) unless otherwise indicated.
C1, cycle 1; Q2W, every 2 weeks; Q2W-LD, cohort receiving a loading dose at Day 1, Cycle 1, followed by a fixed dose every 2 weeks; Q3W, cohort receiving tusamitamab ravtansine every 3 weeks; Q3W, every 3 weeks.
