# Supplementary material for: Tusamitamab Ravtansine in Patients with Advanced Solid Tumors: Phase I Study of Safety, Pharmacokinetics, and Antitumor Activity Using Alternative Dosing Regimens
Source: Cancer Res Commun. 2023 Aug 28;3(8):1662–71. doi: 10.1158/2767-9764.CRC-23-0284 (PMC10461573; doi:10.1158/2767-9764.CRC-23-0284)
Supplement: Supplemental Table S4 — Plasma pharmacokinetics of tusamitamab ravtansine (cohort Q2W-LD) [file crc-23-0284-s06.pdf]

Supplementary Table S4. Plasma pharmacokinetics of tusamitamab ravtansine on Cycle 1 (cohort Q2W-LD)

Tusamitamab ravtansine dose (mg/m²)	*N*	C_{max} (µg/mL)	t_{max}[a] (day)	AUC_{0-14d} (µg•day/mL)	AUC (µg•day/mL)	$t_{1/2z}$ (day)	CL (L/day)	V_{ss} (L)
120	3	53.8 ± 13.7 (52.6) [25]	0.05 (0.04–0.13)	316 ± 92.5 (307) [29]	423 ± 221 (393) [52][b]	8.39 ± 2.61 (8.10) [31]	0.670 ± 0.370 (0.617) [55][b]	5.35 ± 0.985 (5.31) [18][b]
135	4	64.9 ± 23.5 (61.8) [36]	0.20 (0.13–0.33)	334 ± 45.0 (331) [13]	422 ± 60.9 (419) [14][c]	6.30 ± 2.50 (5.92) [40]	0.546 ± 0.118 (0.537) [22][c]	3.74 ± 0.626 (3.71) [17][c]
150	7	90.1 ± 25.2 (87.6) [28]	0.06 (0.05–0.30)	445 ± 40.2 (443) [9]	563 ± 68.2 (560) [12][d]	7.35 ± 1.53 (7.21) [21]	0.526 ± 0.0476 (0.524) [9][d]	4.57 ± 0.782 (4.51) [17][d]
170	12	101 ± 18.5 (99.8) [18]	0.06 (0.05–0.22)	576 ± 162 (555) [28]	773 ± 247 (734) [32][e]	7.13 ± 1.01 (7.06) [14][f]	0.435 ± 0.166 (0.410) [38][e]	4.02 ± 0.951 (3.91) [24][e]

Values are mean ± SD (geometric mean) [CV%] unless otherwise indicated.
[a]Median (min–max); [b]*n* = 2; [c]*n* = 3; [d]*n* = 5; [e]*n* = 10; [f]*n* = 11
AUC, area under the plasma concentration versus time curve from time zero extrapolated to infinity; $AUC_{0–14d}$, area under the plasma concentration versus time curve during the 14-day dosing cycle; CL, clearance; C_{max}, maximum plasma concentration; CV, coefficient of variation; Q2W-LD, cohort receiving a loading dose at Day 1, Cycle 1, followed by a fixed dose every 2 weeks; SD, standard deviation; $t_{½z}$, terminal elimination half-life; t_{max}, time to C_{max}; V_{ss}, volume of distribution at steady state.
