# Supplementary material for: Tusamitamab Ravtansine in Patients with Advanced Solid Tumors: Phase I Study of Safety, Pharmacokinetics, and Antitumor Activity Using Alternative Dosing Regimens
Source: Cancer Res Commun. 2023 Aug 28;3(8):1662–71. doi: 10.1158/2767-9764.CRC-23-0284 (PMC10461573; doi:10.1158/2767-9764.CRC-23-0284)
Supplement: Supplemental Table S5 — Plasma pharmacokinetics of tusamitamab ravtansine (cohort Q3W) [file crc-23-0284-s07.pdf]

Supplementary Table S5. Plasma pharmacokinetics of tusamitamab ravtansine on Cycle 1 (cohort Q3W)

Tusamitamab ravtansine dose (mg/m²)	*N*	C_{max} (µg/mL)	t_{max}[a] (day)	$AUC_{0\text{-}21d}$ (µg•day/mL)	AUC (µg•day/mL)	$t_{1/2z}$ (day)	CL (L/day)	V_{ss} (L)
120	3	66.6 ± 8.7 (66.3) [13]	0.05 (0.04–0.20)	394 ± 55.1 (392) [14]	452 ± 90.9 (445) [20]	6.88 ± 1.94 (6.67) [28]	0.489 ± 0.143 (0.475) [29]	4.02 ± 0.908 (3.96) [23]
150	2	76.6 ± 14.5 (75.7) [19]	0.22 (0.06–0.30)	441 ± 167 (419) [38]	606 ± 200 (589) [33][b]	7.70 ± 1.40 (7.63) [18][b]	0.502 ± 0.181 (0.485) [36][b]	4.81 ± 0.736 (4.79) [15][b]
170	6	84.7 ± 25.2 (81.8) [30]	0.07 (0.05–2.03)	526 ± 139 (510) [26]	590 ± 164 (570) [28]	6.72 ± 0.922 (6.67) [14]	0.532 ± 0.183 (0.506) [34]	4.54 ± 1.67 (4.28) [37]
190	3	98.7 ± 4.59 (98.7) [5]	0.07 (0.06–0.30)	669 ± 126 (661) [19]	775 ± 207 (758) [27]	6.60 ± 0.872 (6.56) [13]	0.431 ± 0.0952 (0.424) [22]	3.68 ± 0.989 (3.60) [27]

Values are mean ± SD (geometric mean) [CV%].

[a]Median (min–max); [b]$n = 2$

AUC, area under the plasma concentration versus time curve from time zero extrapolated to infinity; $AUC_{0–21d}$, area under the plasma concentration versus time curve during the 21-day dosing cycle; CL, clearance; C_{max}, maximum plasma concentration; CV, coefficient of variation; Q3W, cohort receiving tusamitamab ravtansine every 3 weeks; SD, standard deviation; $t_{½z}$, terminal elimination half-life; t_{max}, time to C_{max}; V_{ss}, volume of distribution at steady state.
